# Maternal lifestyle risk factors and human embryonic morphologic development using 3D ultrasound: a population-based prospective cohort study

**DOI:** 10.1093/humrep/deag125

**Published:** 2026-08-14

**Authors:** Naomi Graafland, Anke G Posthumus, Annemarie G M G J Mulders, Romy Gaillard, Anton H J Koning, Vincent W V Jaddoe, Eric A P Steegers, Melek Rousian

**Affiliations:** The Generation R Study Group, Erasmus MC, University Medical Center, Rotterdam, The Netherlands; Department of Obstetrics and Gynecology, Erasmus MC, University Medical Center, Rotterdam, The Netherlands; The Generation R Study Group, Erasmus MC, University Medical Center, Rotterdam, The Netherlands; Department of Obstetrics and Gynecology, Erasmus MC, University Medical Center, Rotterdam, The Netherlands; The Generation R Study Group, Erasmus MC, University Medical Center, Rotterdam, The Netherlands; Department of Obstetrics and Gynecology, Erasmus MC, University Medical Center, Rotterdam, The Netherlands; The Generation R Study Group, Erasmus MC, University Medical Center, Rotterdam, The Netherlands; Department of Pediatrics, Erasmus MC, University Medical Center, Rotterdam, The Netherlands; The Generation R Study Group, Erasmus MC, University Medical Center, Rotterdam, The Netherlands; Department of Obstetrics and Gynecology, Erasmus MC, University Medical Center, Rotterdam, The Netherlands; Department of Pathology, Erasmus MC, University Medical Center, Rotterdam, The Netherlands; The Generation R Study Group, Erasmus MC, University Medical Center, Rotterdam, The Netherlands; Department of Pediatrics, Erasmus MC, University Medical Center, Rotterdam, The Netherlands; The Generation R Study Group, Erasmus MC, University Medical Center, Rotterdam, The Netherlands; Department of Obstetrics and Gynecology, Erasmus MC, University Medical Center, Rotterdam, The Netherlands; Department of Obstetrics and Gynecology, Erasmus MC, University Medical Center, Rotterdam, The Netherlands

**Keywords:** embryo development, environmental effects, epidemiology, pregnancy, ultrasound

## Abstract

**STUDY QUESTION:**

Are periconception maternal modifiable lifestyle factors (i.e. smoking, alcohol consumption, caffeine intake, folic acid supplement use, and physical activity) associated with embryonic morphologic development and growth in a large population-based cohort study?

**SUMMARY ANSWER:**

Inadequate folic acid supplement use and insufficient serum folate levels were associated with delayed embryonic morphologic development and reduced growth, while no associations were observed for smoking, alcohol, caffeine intake, or physical activity.

**WHAT IS KNOWN ALREADY:**

Modifiable maternal lifestyle factors during the periconception period may influence embryonic growth and development. Prior studies suggest associations between smoking, folic acid use, and first trimester growth, but evidence on morphologic development remains limited.

**STUDY DESIGN, SIZE, DURATION:**

This study was embedded in the Generation R Next Study, a population-based periconception cohort in Rotterdam, the Netherlands, conducted between 2017 and 2021. Of the 2173 included spontaneous singleton pregnancies, 993 had reliable pregnancy dating, 3D ultrasound data up to 10 + 2 weeks of gestation, and available information on periconception lifestyle risk factors.

**PARTICIPANTS/MATERIALS, SETTING, METHODS:**

Embryonic development and growth were assessed *in vivo* at 7 and 9 weeks of gestation using 3D transvaginal ultrasound and virtual reality techniques. Morphologic development was determined using the Carnegie staging system based on limb position, embryonic curvature, and brain morphology; crown-rump length (CRL) was used to assess growth. Periconception lifestyle factors, including smoking, alcohol and caffeine intake, physical activity, and folic acid supplement use, were assessed using early pregnancy questionnaires. Linear mixed models were used to examine the association between lifestyle factors and embryonic Carnegie stages and CRL. Analyses were adjusted for gestational age at ultrasound, maternal age, ethnicity, education level, parity, fetal sex, and the other lifestyle factors.

**MAIN RESULTS AND THE ROLE OF CHANCE:**

Inadequate folic acid supplement use was associated with a 0.51-day delay in embryonic development and a 0.72-mm reduction in CRL (−1.6%) at 10 + 2 weeks of gestation. Insufficient serum folate levels (<25.5 nmol/l) were associated with a 1.01-day developmental delay and a 1.24-mm reduction in CRL (−3.7%). No significant associations were observed for smoking, alcohol or caffeine intake, or physical activity. Results remained robust after adjustment for confounders.

**LIMITATIONS, REASONS FOR CAUTION:**

Lifestyle exposures were self-reported, which may have introduced recall or social desirability bias. Gestational age determination was based on the last menstrual period, which can result in dating discrepancies due to variability in cycle length or inaccurate recall, but strict inclusion criteria were applied to minimize misclassification. The relatively healthy study population could limit the generalizability of our results to higher-risk groups.

**WIDER IMPLICATIONS OF THE FINDINGS:**

Our findings emphasize the importance of initiating folic acid supplementation in the preconception period to ensure sufficient maternal folate levels, which may support optimal embryonic morphologic development and growth during the first trimester. These results reinforce public health recommendations advocating timely folic acid supplementation to promote healthy early embryonic development.

**STUDY FUNDING/COMPETING INTEREST(S):**

The Generation R Next Study is financially supported by the Erasmus University Medical Centre Rotterdam, the Erasmus University Rotterdam, and the Netherlands Organization for Health Research and Development. R.G. received funding from the Netherlands Organization for Health Research and Development (NWO, ZonMw VIDI 09150172110034, and NWO, ZonMW, grant number 05430052110007) and an European Research Council Starting Grant (ERC-2024-STG 101161004). V.W.V.J. received a grant from the Netherlands Organization for Health Research and Development (NWO, ZonMw 05430052110007) and a European Research Council Consolidator Grant (ERC-2014-CoG-648916). The authors declare no conflicts of interest.

**TRIAL REGISTRATION NUMBER:**

N/A.

## Introduction

The increasing prevalence of poor nutrition and lifestyle choices poses a significant public health concern. Modifiable lifestyle factors including smoking, alcohol consumption, and lack of physical activity are major contributors to chronic diseases like obesity and type 2 diabetes, cardiovascular conditions, and certain cancers (Chiuve *et al.*, 2008; Loef and Walach, 2012; Altobelli *et al.*, 2020; Li *et al.*, 2020; Oster and Chaves, 2023). This issue is particularly concerning for women of reproductive age, because poor lifestyle choices pose additional risks specifically in pregnancy, impacting fetal growth, development, and the long-term health of the child (Barker, 1999, 2007; Fleming *et al.*, 2018).

Emerging evidence suggests that lifestyle factors in the periconception period, from 14 weeks before conception up to and including the first 10 weeks of gestation, can influence embryonic size and growth, measured in crown-rump length (CRL), and subsequent birth outcomes and childhood health (Mook-Kanamori *et al.*, 2010; van Uitert *et al.*, 2013; Jaddoe *et al.*, 2014; Van Dijk *et al.*, 2018; Oostingh *et al.*, 2019). A growing body of evidence suggests that smoking is associated with a smaller CRL compared with non-smoking women (Mook-Kanamori *et al.*, 2010; Prabhu *et al.*, 2010; van Uitert *et al.*, 2013; Van Dijk *et al.*, 2018; Stamatopoulos *et al.*, 2022), whereas preconceptional initiation of folic acid supplement use has been associated with increased CRL compared with postconceptional initiation or no supplement use (Mook-Kanamori *et al.*, 2010; van Uitert *et al.*, 2013; Van Dijk *et al.*, 2018). Evidence regarding the association between alcohol consumption, caffeine consumption, and physical activity and embryonic growth remains unclear (Mills *et al.*, 1993; Bakker *et al.*, 2010; van Uitert *et al.*, 2013; Van Dijk *et al.*, 2018; van der Windt *et al.*, 2024).

Recent advancements in high-resolution 3-dimensional (3D) ultrasound combined with innovative virtual reality (VR) techniques, now offer real depth perception and a more sensitive assessment of embryonic size (including CRL) and morphologic development. The century-old Carnegie staging system, a measure of human morphologic development, divides the embryonic period into 23 stages based on both internal and external characteristics. Originally developed based on *ex vivo* human embryos, 3D ultrasound in combination with VR now also allows *in vivo* assessment (Rousian *et al.*, 2018). This comprehensive approach considers the entire embryo, rather than just size alone, and therefore potentially provides a more accurate proxy for embryonic health. Only within two previously published studies, embryonic morphology was assessed in relation to periconceptional smoking and levels of red blood cell (RBC) folate, an indicator of folic acid supplement use and dietary intake. The studies were conducted within the Rotterdam Periconception Cohort, a tertiary hospital-based cohort study. These studies concluded that both periconceptional smoking and low RBC folate levels were associated with delayed embryonic development (Parisi *et al.*, 2017; Pietersma *et al.*, 2022).

Against this background, we aim to investigate associations between periconception maternal modifiable lifestyle factors (i.e. smoking, alcohol consumption, caffeine intake, folic acid supplement use, and physical activity) and embryonic morphologic development and growth, in a large population-based cohort study.

## Materials and methods

### Study design

This study was embedded in a subgroup of the Generation R *Next* Study, a population-based prospective cohort study from the preconception period onward in Rotterdam, the Netherlands, and is part of the Generation R Study Programme (Kooijman *et al.*, 2016; Boxem *et al.*, 2024). Its primary aim is to identify preconception and early-pregnancy determinants of fertility, embryonic development, and childhood outcomes. Eligible participants included women and their partners aged ≥18 years, residing in Rotterdam, and either actively trying to conceive or already pregnant, between 2017 and 2021. While enrollment aimed for inclusion during preconception or early pregnancy, enrollment was allowed until delivery. In total, 33.2% and 52.8% of all inclusions were during the preconception period or in the first trimester of pregnancy, respectively. By the end of the study period, 4033 participant episodes from 3602 unique women were included, leading to 3575 pregnancies of 3197 unique women. Study approval was obtained by the Medical Ethical Committee of the Erasmus Medical Center, University Medical Center, Rotterdam (MEC 2016-589, NL57828.078.16). Written informed consent was obtained from all participating women. Findings were reported following the Strengthening the Reporting of Observational Studies in Epidemiology (STROBE) reporting guideline (von Elm *et al.*, 2007).

### Study population

Although enrollment into the overall Generation R Next cohort was possible until delivery, the current analyses focused exclusively on early pregnancy participation. Therefore, only participants with spontaneous, singleton pregnancies who were recruited preconceptionally or during the first trimester, up to 10 + 2 weeks of gestation, which marks the end of the embryonic period (O’Rahilly and Müller, 1987, 2010), were eligible for inclusion. This resulted in a total of 2173 pregnancies. Miscarriages, terminated pregnancies, and pregnancies resulting in perinatal death were excluded (n = 368). Gestational age was calculated from the first day of the last menstrual period (LMP), as CRL, which is used to estimate gestational age in clinical practice, denominated a primary outcome of this study, and doesn’t reflect embryonic growth variation. Pregnancies were excluded if they had an unknown LMP, an irregular cycle (<21 and >35 days), or if there was a difference of more than 6 days between the gestational age based on LMP and the gestational age based on CRL (n = 612). Pregnancies were excluded if participants moved out of the study area, withdrew consent or were lost to follow-up (n = 78). Pregnancies were also excluded if both 3D Carnegie stage and 3D CRL measurement were missing between 6 + 0 and 10 + 2 weeks of gestation (n = 121). This resulted in a total study population of 993 pregnancies, recruited either preconceptionally (29.6%) or in the first trimester of pregnancy (70.4%) (Fig. 1).

**Figure 1.**
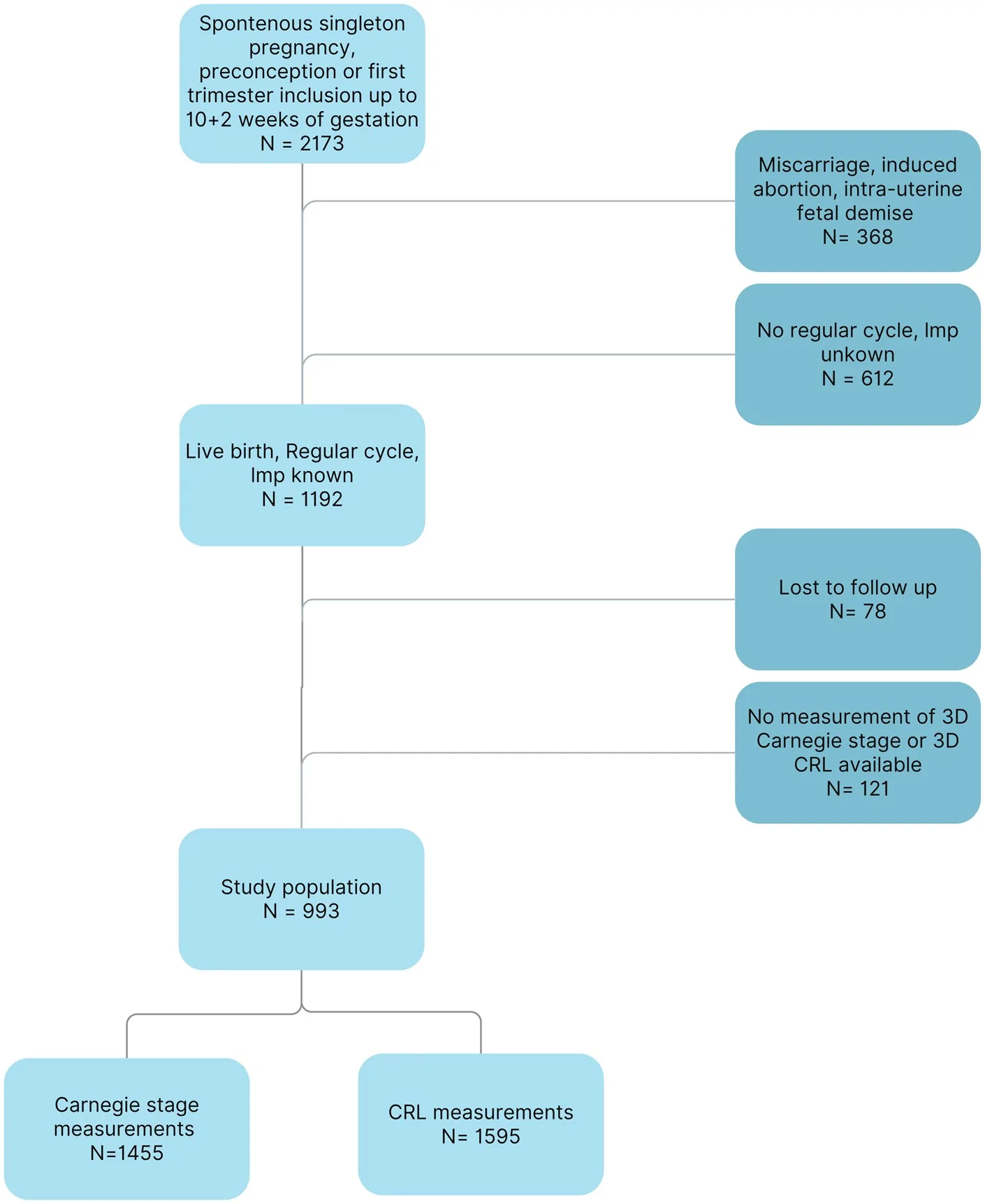
**Flowchart of participants included in the study.** LMP, last menstrual period; CRL, crown-rump length.

### Lifestyle risk factors

The included maternal modifiable lifestyle risk factors (smoking, alcohol consumption, caffeine intake, folic acid supplement use, and physical activity) were selected based on previous research (Graafland *et al.*, 2025) and were assessed using questionnaires administered in early pregnancy.

#### Periconceptional smoking

Women reported on smoking during preconception (3 months before pregnancy) and during pregnancy in the first trimester. Additional information was collected on whether smoking was discontinued and, if so, when during the periconceptional period. For analysis, periconceptional smoking was categorized as no (no smoking or smoking discontinued within 3 months before conception) or yes (smoking during pregnancy). Women who discontinued smoking after conception but within the first trimester were classified as having smoked during pregnancy, reflecting smoking exposure at or around the time of conception.

#### Periconceptional alcohol consumption

Women reported alcohol use during preconception and during pregnancy in the first trimester. Response options included no alcohol use, alcohol use that was discontinued within the 3 months before conception, or alcohol use during pregnancy. For analysis, alcohol consumption was categorized as no (no alcohol use or alcohol use only preconceptionally) or yes (alcohol use during pregnancy), reflecting alcohol exposure at or around the time of conception.

#### Caffeine consumption

Women who reported caffeine intake in pregnancy were asked to specify the type of caffeinated beverages consumed (coffee, tea, cola, or energy drinks) and the daily amount. Total intake was calculated using standard reference values for caffeine content in the Netherlands (Voedingscentrum, 2022). One unit was defined as ∼60 mg of caffeine, corresponding to one standard cup of coffee. Caffeine consumption was subsequently categorized as none, <2 units/day, 2–3.9 units/day, or ≥4 units/day.

#### Physical activity

Participants were asked to report the amount of time spent per week in moderate and strenuous physical activity. Moderate physical activity was defined in the questionnaire as activities during which participants were generally still able to talk, but with increased heart rate and breathing (e.g. brisk walking, cycling at <20 km/h, or leisurely swimming). Strenuous physical activity was defined as activities during which participants were unable to speak more than a few words consecutively due to markedly increased breathing and heart rate (e.g. running, cycling at >20 km/h, fast swimming, or intensive gardening). Physical activity was then categorized as not active, moderately active (<150 min of moderate activity per week or <75 min of strenuous activity per week), or very active (>150 min of moderate activity per week or >75 min of strenuous activity per week). A very active lifestyle was used as the reference category, as maintaining an active lifestyle is recommended during the periconception period (ACOG, 2020).

#### Folic acid supplement use

In accordance with the recommendation to initiate folic acid supplement use preconceptionally, we categorized folic acid supplement use in two groups: adequate use (preconceptional initiation of supplement use) and inadequate use (postconceptional initiation of supplement use or no use at all) (Centers for Disease Control, 1993; World Health Organization, 2007).

### Maternal serum folate concentrations

As a key biomarker complementing folic acid supplement use, serum folate concentrations were also assessed. Maternal serum samples were collected during the first prenatal visit in early pregnancy (median 7 + 5 weeks; 90% range 6 + 5 to 9 + 2 weeks). Folate concentrations in these samples were determined using an immunoelectrochemoluminescence assay. The upper limit of detection (LOD) of folate was 45.4 nmol/l and any concentration above this limit was imputed as 45.4 nmol/l. Serum folate concentrations were categorized as sufficient (≥25.5 nmol/l) or insufficient (<25.5 nmol/l), based on recommendations for women of reproductive age (Cordero *et al.*, 2015; Chen *et al.*, 2019).

### Outcomes

During the first trimester, transvaginal 3D ultrasound volume sweeps of the embryo were performed by trained sonographers following cohort-specific guidelines (Campbell and Wilkin, 1975; Verburg *et al.*, 2008; Ioannou *et al.*, 2013; Bilardo *et al.*, 2023). Sonographers were trained following national standards set by the Dutch Ministry of Public Health, Welfare, and Sports; thus, measurement variability was assumed to be representative of clinical practice in the Netherlands. Ultrasound examinations were carried out using the Voluson© E10 (GE Healthcare), Philips© Affiniti 70, or Philips© Epiq 7 systems. Transvaginal transducers with varying frequency ranges (4.0–9.0 MHz or 4.5–11.9 MHz) were selected according to the study protocol, primarily based on gestational age and optimal visualization of embryonic structures, with higher-frequency probes more commonly used in earlier gestations. If initial image quality was insufficient, the 3D volume sweep was repeated using an alternative transducer. Routine quality control procedures were performed to monitor image quality throughout the study period. At each ultrasound examination, two replicate 3D embryonic volume sweeps were acquired to ensure adequate image quality for subsequent analysis.

To enable offline analysis, all 3D ultrasound data were converted into Cartesian (rectangular) volumes. In-house developed V-Scope software (Erasmus MC, Rotterdam, the Netherlands) (Koning *et al.*, 2009) was used to generate embryonic holograms on a VR system named the I-Wall (Rousian *et al.*, 2018). This is a flat 3D rear-projection-based semi-immersive VR system, offering real depth perception using stereoscopic glasses. For this study, embryonic morphological development and embryonic growth were assessed from the offline 3D ultrasound data acquired during the embryonic period, defined as gestational ages ≤10 + 2 weeks. Therefore, although ultrasound examinations were scheduled at ∼7, 9, and 11 weeks of gestation, only scans within this gestational age window were included in the analyses. Carnegie stages were determined in 967 pregnancies, with 50.4% (488 pregnancies) assessed at two timepoints. Similarly, 3D CRL measurements were performed in 991 pregnancies, with 60.9% (604 pregnancies) evaluated at two timepoints. The availability of one or two scans during the embryonic period varied due to later inclusion in the study, missed appointments, gestational age at the time of the ultrasound exceeding the predefined study period (≥10 + 3 weeks), participant preference not to undergo transvaginal 3D ultrasound and insufficient image quality to allow reliable offline measurements.

Embryonic morphologic development was assessed using the Carnegie staging system. This system was originally designed based on *ex vivo* human embryos, using internal, external, and histologic criteria. VR enables the *in vivo* determination of these stages, based on the following criteria: position of upper and lower limbs, curvature of the embryo, and morphological features of the pros-, mes-, and rhombencephalon (O’Rahilly and Müller, 2010). Morphologic development was visually compared to the Carnegie stages 13–23, as refined by O’Rahilly and Müller in 1987 (O’Rahilly and Müller, 1987, 2010; Verwoerd-Dikkeboom *et al.*, 2008a; Rousian *et al.*, 2018; Flierman *et al.*, 2023; Pietersma *et al.*, 2023). Figure 2 shows representative examples of embryos at Carnegie stages 15, 17, 20, and 23. Before initiating measurements, assessors completed a structured training and learning period. Measurements were only performed after excellent reproducibility was achieved (ICC > 0.90). Embryonic growth was assessed using CRL measurements, the length from the cranium to the caudal rump in a straight line within the mid-sagittal plane (Robinson, 1973; Robinson and Fleming, 1975). Using VR techniques, very precise caliper positioning is possible (Verwoerd-Dikkeboom *et* *al.*, 2008b).

**Figure 2.**
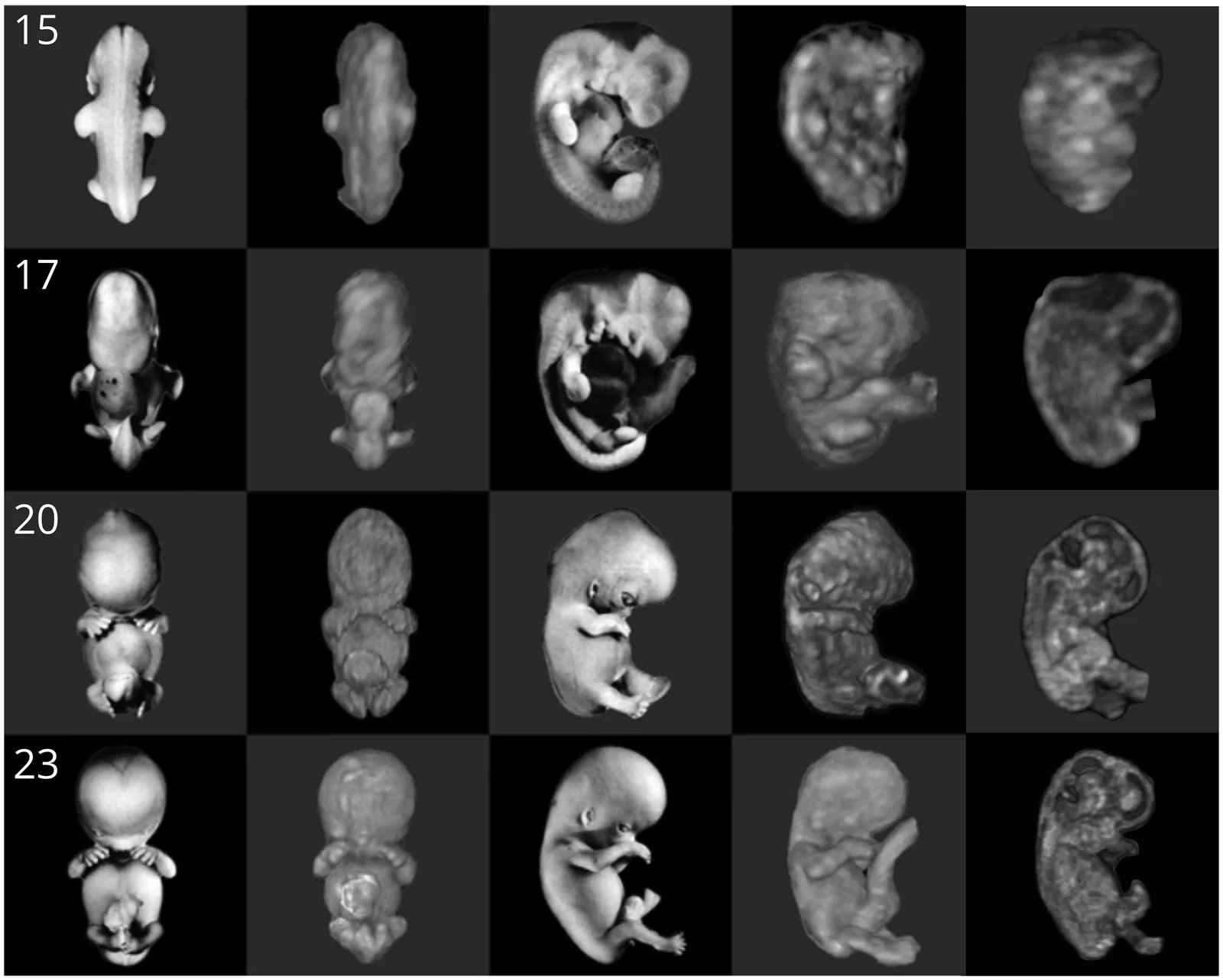
**Comparison of embryos at four different Carnegie stages from the original Carnegie collection and *in vivo* embryos assessed using 3D ultrasound and virtual reality.** Images are shown in the dorsal, sagittal, and midsagittal planes. The numbers in the left-hand panels indicate the Carnegie stages, assigned based on the assessment of internal and external morphological features of the embryo, including the development and position of the limbs, curvature of the embryo, and morphological features of the prosencephalon, mesencephalon, and rhombencephalon.

### Covariates

Information on maternal age at conception (years), ethnicity (Dutch; non-Dutch, European; non-Dutch, non-European), educational level (low: no or primary education; medium: secondary education; high: university degree), and parity (nulliparous; parous) was obtained through questionnaires at enrollment. Fetal sex and birth weight were obtained from midwifery registries and obstetric medical records.

### Statistical analyses

First, we conducted a nonresponse analysis, comparing participants that were included in our study to those that were excluded due to an unknown or an unreliable LMP, or an irregular menstrual cycle by using the *t*-test, the Mann–Whitney *U*-test, or the χ^2^ test. To account for selection bias by irregular or unknown cycle, inverse probability weighting was performed, by using information available from both groups (i.e. irregular or unknown cycle and known cycle) to predict the probability of participation in the study. The inverse of those probabilities was used as weights in the analyses to ensure representative results for the initial cohort population (Weuve *et al.*, 2012; Weisskopf *et al.*, 2015). Second, linear mixed models (LMM) were used to examine the associations between each modifiable lifestyle risk factor (i.e. periconception smoking, alcohol consumption, caffeine consumption, physical activity, and folic acid supplement use) and longitudinally measured 3D Carnegie stages and CRL. To improve interpretability, additional analyses were performed for lifestyle factors that showed an association with Carnegie stage or CRL. Linear mixed models including interaction terms between the lifestyle factor and gestational age were fitted. For Carnegie stage analyses, the interaction model was used to estimate the gestational age at which Carnegie stage 23 (representing the end of embryonic development) was reached for exposed and unexposed pregnancies; the difference between these estimates was expressed as a delay in days. For CRL analyses, estimated CRL values for exposed and unexposed pregnancies were derived at a fixed gestational age of 10 + 2 weeks, and differences were expressed as percentage change. In the crude model (model 1), the association between lifestyle risk factors and Carnegie stage and CRL was adjusted only for gestational age. Model 2 included additional adjustments for potential confounders including maternal age at conception, ethnicity, educational level, parity, and fetal sex. To examine the independent associations of each lifestyle factor with the outcomes, model 3 was further adjusted for the other studied lifestyle risk factors. Third, we conducted a separate analysis to assess the association between serum folate levels and trajectories of Carnegie Stages and CRL. Folate levels were evaluated as both a continuous variable and a categorical variable (sufficient (≥25.5 nmol/l) vs insufficient (<25.5 nmol/l)). The same adjustments were applied across for model 1. Model 2 was additionally adjusted for potential confounders, including maternal age at conception, body mass index (BMI), ethnicity, educational level, parity, fetal sex, and smoking. As a sensitivity analysis, we analyzed a subgroup of the population, excluding the participants with a serum folate above the upper LOD (>45.3 nmol/l), to reduce the influence of extreme outliers. Potential confounders for all analyses were selected *a priori* based on a directed acyclic graph (DAG) and associations observed in existing literature (Supplementary Fig. S1). Missing data for potential confounders were lower than 10%. To limit selection bias and loss of statistical power, multiple imputation was applied. A total of 25 complete datasets were obtained and analyzed using standard procedures for multiple imputation (Graham *et al.*, 2007; Sterne *et al.*, 2009). All statistical analyses were performed using R Statistical Software (version 4.3.0; R Foundation for Statistical Computing, Vienna, Austria).

## Results

### Characteristics of the study population

A total of 993 women were included in the study, with 294 recruited preconceptionally and 699 during early pregnancy (up to 10 + 2 weeks of gestation). A total of 1597 3D datasets were collected, enabling 1455 measures of Carnegie stage and 1595 measures of CRL. Baseline characteristics of the study population are presented in Table 1. Women had a mean age at conception of 31.4 years (SD: 3.9) and a median pre-pregnancy BMI of 23.2 kg/m^2^ (IQR: 21.2–26.0). Most women were of Dutch nationality, highly educated, and nulliparous. During the periconception period, 69.5% of women used folic acid supplements adequately, and 85.3% had sufficient serum folate levels. Serum folate concentrations exceeded the assay’s upper LOD in 19.5% of women. Overall 22.2% of women reported smoking in pregnancy; of these, 41.5% discontinued smoking during the first trimester. Additionally, 18.3% pregnant women consumed alcohol, and 8.7% consumed more than 4 units of caffeine daily. Furthermore, 75.2% of women were very physically active during the first trimester. Nonresponse analysis revealed that women with a known LMP and a regular cycle were more likely to be of Dutch nationality, highly educated, have higher monthly household incomes, be smokers, consume alcohol, and engage in more physical activity compared to those with an unknown LMP or an irregular cycle (Supplementary Table S1).

**Table 1.** Baseline characteristics of the total population.

Maternal characteristics	Distribution	Missing
	N = 993	
Maternal age at conception (years)	31.4 (3.9)	0
Pre-pregnancy BMI (kg/m^2^)	23.2 (21.2–26.0)	64 (6.5)
Ethnicity (%)		25 (2.5)
Dutch	665 (67.7)	
Non-Dutch, European	111 (11.5)	
Non-Dutch, non-European	202 (20.9)	
Maternal educational level (%)		32 (3.2)
Low	34 (3.5)	
Medium	201 (20.9)	
High	726 (75.6)	
Monthly household income (%)		108 (10.9)
<2000 euro	68 (7.7)	
2000–4000 euro	285 (32.2)	
>4000 euro	532 (60.1)	
Parity (%)		45 (4.5)
Nulliparous	627 (66.1)	
Parous	321 (33.9)	
First trimester smoking (yes, %)	200 (22.2)	90 (9.1)
First trimester alcohol consumption (yes, %)	175 (18.3)	38 (3.8)
Daily caffeine consumption, units^a^ (%)	111 (55–167)	71 (7.2)
None	50 (5.4)	
<2 units	444 (48.2)	
2–3.9 units	348 (37.7)	
> 4 units	80 (8.7)	
Physical activity (%)		72 (7.3)
Not active	17 (1.9)	
Moderately active (<150 min moderate or <75 min strenuous intensity)	211 (22.9)	
Very active (>150 min moderate or >75 min strenuous intensity)	693 (75.2)	
Folic acid supplement use (inadequate, %)^b^	287 (30.5)	52 (5.2)
Serum folate (nmol/l)	37.7 (30.1–44.7)	112 (11.3)
Sufficient^c^ (≥25.5 nmol/l)	752 (85.3)	
Insufficient (<25.5 nmol/l)	129 (14.7)	
Birth outcomes		
Fetal sex (male, %)	522 (55.6)	0
Gestational age at birth, weeks (IQR)	39.7 (38.7–40.6)	0
Preterm birth (%)	54 (5.4)	0
Birth weight, Hoftiezer percentile (IQR)	0.42 (0.18–0.67)	22 (2.2)

Values are presented as mean (SD), median (IQR), or number of participants (valid %).

a One unit reflects 90 mg caffeine.

b Folic acid supplement use; adequate: preconceptional initiation of supplement use, inadequate: postconceptional initiation of supplement use or no use at all.

c Sufficient levels ≥25.5 nmol/l serum folate, based on recommendations for women of reproductive age.

### Lifestyle risk factors and embryonic development

Table 2 shows the associations between maternal periconception modifiable lifestyle risk factors and morphologic development according to the Carnegie stages. Linear mixed models showed that inadequate folic acid supplement use was associated with lower than expected values of the Carnegie stages, compared with adequate preconceptional supplementation (β −0.24 [95% CI: −0.39 to −0.10]). After adjustment for covariates and other lifestyle risk factors, the associations persisted with comparable effect estimates. Essentially, this indicates that embryo’s from mothers with inadequate folic acid supplementation reach the final Carnegie stage with a delay of 0.51 days compared to those with adequate supplementation. No associations were observed between periconception smoking, alcohol consumption, caffeine consumption, and physical activity and embryonic morphologic development according to the Carnegie stages.

**Table 2.** Association between periconceptional lifestyle risk factors and embryonic development (Carnegie stages) and growth (crown-rump length).

	Carnegie stage (beta (95% CI))			Crown-rump length (beta (95% CI))		
	Model 1^a^	Model 2^b^	Model 3^c^	Model 1^a^	Model 2^b^	Model 3^c^
Periconception smoking		*N = 879*			*N = 901*	
No	*Reference*	*Reference*	*Reference*	*Reference*	*Reference*	*Reference*
Yes	−0.06 (−0.28 to 0.14)	−0.06 (−0.27 to 0.15)	−0.03 (−0.24 to 0.19)	−0.19 (−0.75 to 0.36)	−0.19 (−0.48 to 0.10)	−0.16 (−0.46 to 0.14)
Periconception alcohol consumption		*N = 929*			*N = 953*	
No	*Reference*	*Reference*	*Reference*	*Reference*	*Reference*	*Reference*
Yes	0.06 (−0.11 to 0.23)	0.02 (−0.15 to 0.20)	0.04 (−0.13 to 0.22)	0.20 (−0.29 to 0.68)	−0.05 (−0.29 to 0.18)	−0.01 (−0.25 to 0.22)
Daily caffeine consumption		*N = 897*			*N = 920*	
None	*Reference*	*Reference*	*Reference*	*Reference*	*Reference*	*Reference*
<2 units	−0.13 (−0.44 to 0.18)	−0.20 (−0.51 to 0.11)	−0.25(−0.56 to 0.07)	−0.25 (−1.12 to 0.62)	−0.13 (−0.56 to 0.30)	−0.17 (−0.60 to 0.26)
2–3.9 units	−0.14 (−0.45 to 0.18)	−0.21 (−0.52 to 0.11)	−0.26 (−0.58 to 0.06)	−0.27 (−1.15 to 0.61)	−0.19 (−0.63 to 0.25)	−0.23 (−0.67 to 0.21)
≥4 units	−0.15 (−0.53 to 0.22)	−0.21 (−0.59 to 0.17)	−0.28 (−0.67 to 0.10)	−0.42 (−1.47 to 0.63)	−0.20 (−0.72 to 0.33)	−0.26 (−0.78 to 0.27)
Phsyical activity		*N = 896*			*N = 919*	
Not active	0.35 (−0.18 to 0.87)	0.41 (−0.13 to 0.95)	0.38 (−0.16 to 0.93)	1.53 (0.11–2.95)*	0.72 (−0.02 to 1.47)	0.72 (−0.02 to 1.47)
Moderately active	−0.07 (−0.23 to 0.09)	−0.06 (−0.22 to 0.11)	−0.05 (−0.21 to 0.12)	−0.12 (−0.56 to 0.42)	0.14 (−0.08 to 0.37)	0.15 (−0.08 to 0.37)
Very active	*Reference*	*Reference*	*Reference*	*Reference*	*Reference*	*Reference*
Folic acid supplement use^d^		*N = 917*			*N = 939*	
Adequate	*Reference*	*Reference*	*Reference*	*Reference*	*Reference*	*Reference*
No or inadequate	−0.24 (−0.39 to −0.10)*	−0.24 (−0.39 to −0.08)*	−0.25 (−0.41 to −0.09)*	−0.46 (−0.87 to −0.05)*	−0.23 (−0.44 to −0.02)*	−0.23 (−0.44 to −0.02)*

a Model 1: GA.

b Model 2: GA, maternal age at conception, ethnicity, educational level, parity, and fetal sex.

c Model 3: GA, maternal age at conception, ethnicity, educational level, parity, fetal sex, periconception smoking, alcohol consumption in first trimester, caffeine consumption, physical activity, and folic acid supplement use (unless exposure of interest).

d Folic acid supplement use; adequate: preconceptional initiation of supplement use, inadequate: postconceptional initiation of supplement use or no use at all.

* *P*-value <0.05.

GA, gestational age.

### Serum folate and embryonic development

Table 3 shows the associations between maternal periconception serum folate levels and Carnegie stages. A higher serum folate level was associated with accelerated embryonic development (β 0.21 [95% CI: 0.09–0.32], per IQR (14.6 nmol/l) increase in serum folate). Moreover, an insufficient serum folate level in the periconception period was associated with a 1.01-day delay in reaching the final Carnegie stage, compared to a sufficient serum folate level (Fig. 3). After excluding participants with a serum folate level above the upper LOD from all analysis, the effect estimates remained comparable (Supplementary Table S2).

**Figure 3.**
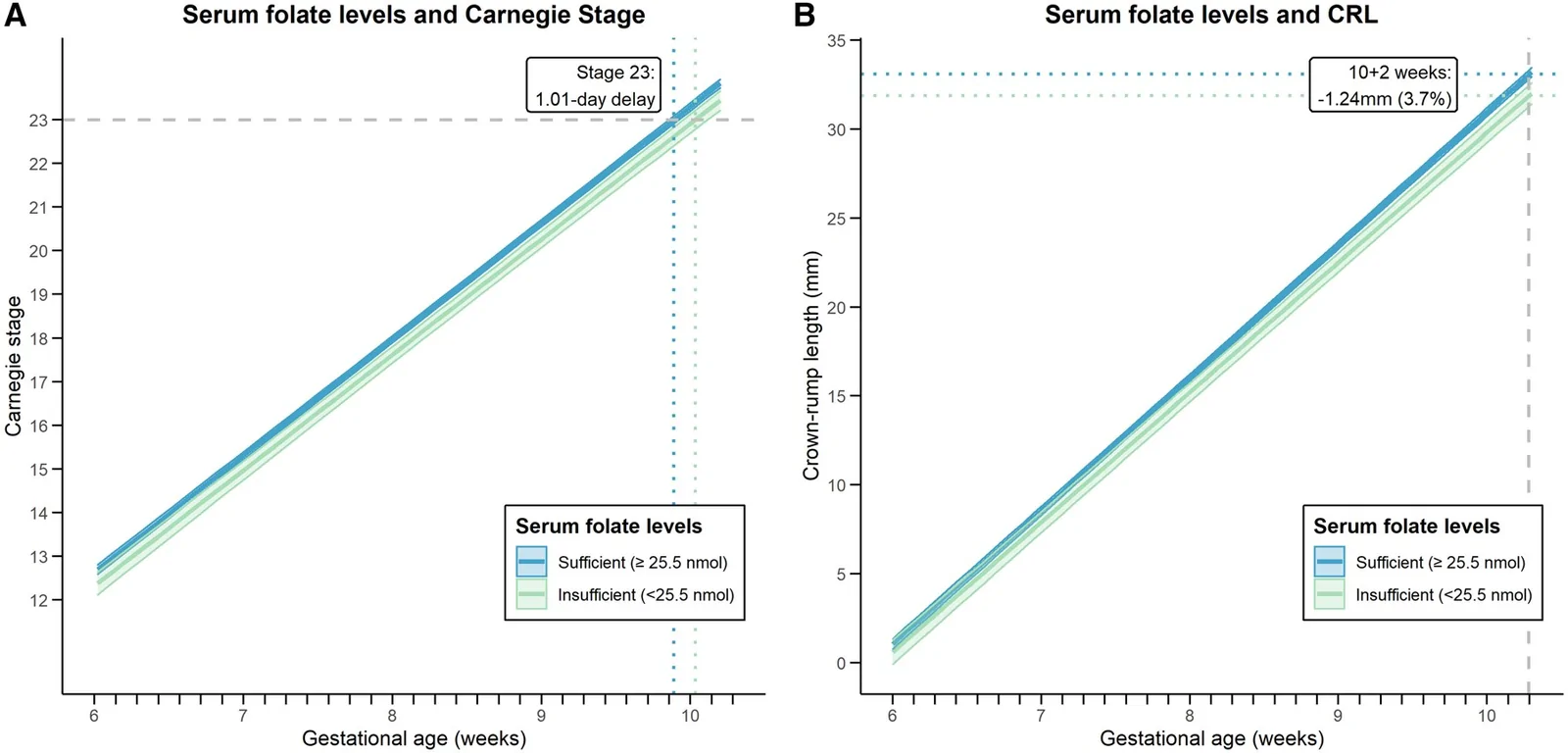
**Effect plots of differences in embryonic outcomes.** The association of sufficient folate levels is shown for embryonic development (**A**) and crown-rump length (**B**). The reported delay (in A) refers to the time taken to reach the final Carnegie stage (Stage 23) between groups with sufficient and insufficient folate levels. The reported percentages (in B) represent the differences in estimated CRL between groups with sufficient and insufficient folate levels. Insufficient plasma folate levels are defined as below the recommended threshold for women of reproductive age (25.5 nmol/l). CRL, crown-rump length.

**Table 3.** Association between periconceptional lifestyle risk factors and embryonic development (Carnegie stages) and growth (crown-rump length).

	Carnegie stage (beta (95% CI)) *N = 808*		Crown-rump length (beta (95% CI)) *N = 828*	
	Model 1^a^	Model 2^b^	Model 1^a^	Model 2^b^
Serum folate (per IQR increase = 14.6 nmol/l)	0.21 (0.09–0.32)*	0.19 (0.06–0.32)*	0.48 (0.17–0.80)*	0.51 (0.15–0.86)*
Serum folate				
Sufficient^c^ (≥25.5 nmol/l)	*Reference*	*Reference*	*Reference*	*Reference*
Insufficient (<25.5 nmol/l)	−0.36 (−0.56 to −0.16)*	−0.33 (−0.55 to −0.11)*	−0.89 (−1.45 to −0.33)*	−0.93 (−1.55 to −0.32)*

a Model 1: GA.

b Model 2: GA, maternal age at conception, BMI, ethnicity, educational level, parity, smoking and fetal sex consumption, physical activity, and folic acid supplement use (unless exposure of interest).

c Sufficient levels ≥25.5 nmol/l serum folate, based on recommendations for women of reproductive age.

* *P*-value <0.05.

IQR, interquartile range; GA, gestational age.

### Lifestyle risk factors, serum folate levels, and CRL

Linear mixed models showed that inadequate folic acid supplement use was associated with a smaller CRL (−0.46 mm [95% CI: −0.87 to −0.05]) compared with adequate folic acid supplement use (Table 2). Essentially, this indicates a reduction in CRL of −0.72 mm (−1.6%) at 10 + 2 weeks of gestation. Additionally, a higher serum folate level was associated with larger CRL (0.48 mm [95% CI: 0.17–0.80], per IQR (= 14.6 nmol/l) increase in serum folate) and a periconception insufficient serum folate level was associated with a reduction in CRL of 1.24 mm (−3.7%) at 10 + 2 weeks, compared with a sufficient serum folate level (Fig. 3). No associations were observed between periconception smoking, alcohol consumption, caffeine consumption, and embryonic growth. Additionally, in Model 1, a non-active lifestyle, compared to a very active lifestyle, was positively associated with CRL (β  =  1.53 mm [95% CI: 0.11–2.95]). After adjustment for confounders, particularly maternal age at conception, this association was no longer statistically significant.

## Discussion

### Main findings

This study investigated the association between periconceptional lifestyle risk factors and embryonic morphological development, as assessed by the Carnegie stages, in a large population-based periconception cohort. We found that no or inadequate folic acid supplementation and insufficient serum folate levels were associated with delayed embryonic development and reduced embryonic growth. No associations were demonstrated between smoking, alcohol consumption, caffeine consumption, and physical activity and embryonic development and growth.

### Interpretation of findings and comparison with the literature

Our findings on serum folate and embryonic development are in line with only one previously conducted study. This study was performed within a prospective cohort study, including 234 high-risk pregnancies, and reported that low RBC folate concentrations were associated with delayed embryonic development in IVF/ICSI pregnancies (Parisi *et al.*, 2017). RBC folate levels indicate long-term folate status, whereas serum folate is considered an indicator of more recent folate intake(World Health Organization, 2015). Although serum folate levels can fluctuate based on recent dietary intake, RBC folate measurement is more complex and costly. Therefore, it is relevant that our study observed the same association with serum folate, a more commonly used marker in clinical practice and large epidemiological studies. This makes serum folate a practical and accessible measure for assessing folate-related risks in the periconception period. No prior studies have investigated the association between folic acid supplement use and embryonic development. However, our findings do align with the majority of the studies on folic acid supplement use and growth assessed by CRL, showing a positive association with adequate periconception supplement use (Mook-Kanamori *et al.*, 2010; van Uitert *et al.*, 2013; Van Dijk *et al.*, 2018). Several possible explanations exist for the effect of folic acid supplement use and serum folate on embryonic development and growth rate. First, folate plays a key role in one-carbon metabolism, providing essential one-carbon groups for critical processes such as DNA synthesis, protein synthesis, and epigenetic programming (Crider *et al.*, 2012; Steegers-Theunissen *et al.*, 2013). These processes are vital during early pregnancy, a period characterized by rapid cell division, growth, and high sensitivity to external influences and deficiencies may impair epigenetic programming, potentially leading to long-term health consequences for the child. Another potential mechanism involves the mechanistic target of rapamycin (mTOR), a folate sensor in the trophoblast cells important for placental amino acid transport and mitochondrial function (Rosario *et al.*, 2016, 2017b; Silva *et al.*, 2017). Folate deficiency has been shown to inhibit mTOR signaling, resulting in downregulation of placental amino acid transporters and impaired trophoblast function, which can restrict embryonic and fetal development (Yung *et al.*, 2008; Roos *et al.*, 2009; Chen *et al.*, 2015; Rosario *et al.*, 2017a).

In the present study, which included only spontaneous pregnancies, no association was found between smoking and embryonic development. In contrast, a previous prospective cohort study of 689 women in a high-risk population reported an inverse association between smoking and embryonic development (Pietersma *et al.*, 2022). Notably, nearly half (49.2%) of that population consisted of IVF/ICSI pregnancies, and after stratifying by mode of conception, the effect of smoking was observed only in the IVF/ICSI group. This may be due to the precise dating of IVF/ICSI pregnancies, which allows for more accurate detection of small effects. Additionally, IVF/ICSI pregnancies may be more susceptible to external exposures due to the techniques involved in assisted reproduction (Klonoff-Cohen, 2005; Berntsen *et al.*, 2019).

Alcohol and caffeine consumption and physical activity were not significantly associated with embryonic development in the present study. No prior studies investigated these associations, but studies that focused on CRL reported inconsistent results for alcohol and caffeine consumption (Mills *et al.*, 1993; Bakker *et al.*, 2010; Mook-Kanamori *et al.*, 2010; van Uitert *et al.*, 2013; Van Dijk *et al.*, 2018). Regarding caffeine, one large cohort study among 1310 healthy pregnancies demonstrated an association between caffeine consumption of more than 540 mg/day (>6 units) and smaller CRL (Bakker *et al.*, 2010). In the present study, the maximum reported caffeine consumption was 4 units per day, which may explain why we did not observe any significant associations. Regarding periconception alcohol consumption, only one small prospective cohort study (n = 77) in a high-risk population showed a negative association with CRL (van Uitert *et al.*, 2013), whereas other studies found no association (Van Dijk *et al.*, 2018; van der Windt *et al.*, 2024).

### Strengths and limitations

A key strength of this study is its large, population-based design, with many spontaneous pregnancies included in the periconception period. Additionally, the availability of serial 3D ultrasound data during the embryonic period is a unique feature as well. The 3D VR approach allowed for detailed observation of the embryonic morphology and an accurate and reliable CRL measurement. A potential limitation of this study is that pregnancy dating was based on the LMP, which is not the preferred method in clinical practice and can be less accurate due to variability in cycle length or inaccurate recall of the LMP (Savitz *et al.*, 2002; Mahendru *et al.*, 2012). However, to reduce the potential for gestational age misclassification, we only included pregnancies with regular cycles and a known LMP. Most of the lifestyle risk factors were explored using self-reported questionnaires, introducing the possibility of social desirability and recall bias and thus the chance of underreporting. This could result in an underestimation of the true effects of these risk factors. The included study population was relatively healthy, characterized by low exposure to adverse lifestyle factors, high adherence to periconceptional folic acid supplementation, and a high socioeconomic status. This may limit the generalizability of the findings to higher-risk group and additionally could make it more challenging to detect the effects of certain lifestyle risk factors. Finally, it is worth noting that the Carnegie staging system is based on *ex vivo* examination of external and internal characteristics. *In vivo*, however, not all these characteristics can be observed, which may limit the precision of embryonic staging.

### Clinical and research implications

The findings of this large population-based cohort study suggest that health and lifestyle factors in the periconception period, particularly folic acid supplementation and serum folate levels, influence embryonic health. This underscores the critical importance of this period as a window for intervention. Although preconception counseling on folic acid supplementation is recommended in current guidelines, timely initiation before conception remains suboptimal in many populations. Our results highlight the importance of not only recommending folic acid use, but also ensuring early initiation and adequate folate levels prior to conception. Population-level strategies aimed at improving access and uptake, such as the provision of free folic acid supplements as implemented in some regions (e.g. Scotland), may support more effective preconception care. Future research should focus on identifying and evaluating strategies to improve timely uptake and adherence to folic acid supplementation before conception and assess whether these approaches translate into improved embryonic development and downstream pregnancy outcomes. Further research should prioritize using the Carnegie staging system in addition to crown-rump length, as it offers a more comprehensive marker of embryonic health. Furthermore, the incorporation of objective measures, such as biomarkers for alcohol (e.g. phosphatidylethanol) and smoking (e.g. cotinine or carbon monoxide testing) (Shipton *et al.*, 2009; Wassenaar and Koch, 2015), alongside activity trackers to accurately monitor physical activity, would help to further elucidate the relationship between lifestyle risk factors and embryonic health and beyond.

In conclusion, inadequate folic acid supplement use and insufficient serum folate levels are associated with delayed embryonic development and reduced growth rates in the early stages of pregnancy. These findings highlight the importance of preconception initiation of folic acid supplementation to support embryonic health, underscoring the critical role of the periconception period. Incorporating embryonic morphologic development alongside growth in future research can provide deeper insights into the pathophysiology of external exposures on embryonic health, helping to determine how and when to intervene to optimize embryonic health and improve birth and childhood outcomes.

## Supplementary Material

deag125_Supplementary_Figure_S1

deag125_Supplementary_Table_S1

deag125_Supplementary_Table_S2

## Data Availability

The data underlying this article will be shared on reasonable request to the corresponding author.

## References

[deag125-B1] ACOG. Physical activity and exercise during pregnancy and the postpartum period: ACOG Committee Opinion, Number 804. Obstet Gynecol 2020;135:178–188. 10.1097/AOG.0000000000003772 32217980

[deag125-B2] Altobelli E , Angeletti PM, Profeta VF, Petrocelli R. Lifestyle risk factors for type 2 diabetes mellitus and national diabetes care systems in European countries. Nutrients 2020;12:2806. 10.3390/nu12092806 PMC7551066 32933175

[deag125-B3] Bakker R , Steegers EAP, Obradov A, Raat H, Hofman A, Jaddoe VWV. Maternal caffeine intake from coffee and tea, fetal growth, and the risks of adverse birth outcomes: the Generation R Study. Am J Clin Nutr 2010;91:1691–1698. 10.3945/ajcn.2009.28792 20427730

[deag125-B4] Barker DJ. Fetal origins of cardiovascular disease. Ann Med 1999;31:3–6. 10.1080/07853890.1999.11904392 28850284

[deag125-B5] Barker DJP. The origins of the developmental origins theory. J Intern Med 2007;261:412–417. 10.1111/j.1365-2796.2007.01809.x 17444880

[deag125-B6] Berntsen S , Söderström-Anttila V, Wennerholm UB, Laivuori H, Loft A, Oldereid NB, Romundstad LB, Bergh C, Pinborg A. The health of children conceived by ART: ‘the chicken or the egg?’. Hum Reprod Update 2019;25:137–158. 10.1093/humupd/dmz001 30753453

[deag125-B7] Bilardo CM , Chaoui R, Hyett JA, Kagan KO, Karim JN, Papageorghiou AT, Poon LC, Salomon LJ, Syngelaki A, Nicolaides KH; International Society of Ultrasound in Obstetrics and Gynecology. ISUOG Practice Guidelines (updated): performance of 11-14-week ultrasound scan. Ultrasound Obstet Gynecol 2023;61:127–143. 10.1002/uog.26106 36594739

[deag125-B8] Boxem AJ , Blaauwendraad SM, Mulders A, Bekkers EL, Kruithof CJ, Steegers EAP, Gaillard R, Jaddoe VWV. Preconception and early-pregnancy body mass index in women and men, time to pregnancy, and risk of miscarriage. JAMA Netw Open 2024;7:e2436157. 10.1001/jamanetworkopen.2024.36157 PMC11413718 39298166

[deag125-B9] Campbell S , Wilkin D. Ultrasonic measurement of fetal abdomen circumference in the estimation of fetal weight. Br J Obstet Gynaecol 1975;82:689–697. 10.1111/j.1471-0528.1975.tb00708.x 1101942

[deag125-B10] Centers for Disease Control. Recommendations for use of folic acid to reduce number of spina bifida cases and other neural tube defects. Jama 1993;269:1233–1238. 8437292

[deag125-B11] Chen M-Y , Rose CE, Qi YP, Williams JL, Yeung LF, Berry RJ, Hao L, Cannon MJ, Crider KS. Defining the plasma folate concentration associated with the red blood cell folate concentration threshold for optimal neural tube defects prevention: a population-based, randomized trial of folic acid supplementation. Am J Clin Nutr 2019;109:1452–1461. 10.1093/ajcn/nqz027 PMC7099800 31005964

[deag125-B12] Chen YY , Rosario FJ, Shehab MA, Powell TL, Gupta MB, Jansson T. Increased ubiquitination and reduced plasma membrane trafficking of placental amino acid transporter SNAT-2 in human IUGR. Clin Sci (Lond) 2015;129:1131–1141. 10.1042/CS20150511 PMC4614027 26374858

[deag125-B13] Chiuve SE , Rexrode KM, Spiegelman D, Logroscino G, Manson JE, Rimm EB. Primary prevention of stroke by healthy lifestyle. Circulation 2008;118:947–954. 10.1161/CIRCULATIONAHA.108.781062 PMC2730914 18697819

[deag125-B14] Cordero AM , Crider KS, Rogers LM, Cannon MJ, Berry RJ. Optimal serum and red blood cell folate concentrations in women of reproductive age for prevention of neural tube defects: World Health Organization guidelines. MMWR Morb Mortal Wkly Rep 2015;64:421–423. PMC5779552 25905896

[deag125-B15] Crider KS , Yang TP, Berry RJ, Bailey LB. Folate and DNA methylation: a review of molecular mechanisms and the evidence for folate’s role. Adv Nutr 2012;3:21–38. 10.3945/an.111.000992 PMC3262611 22332098

[deag125-B16] Fleming TP , Watkins AJ, Velazquez MA, Mathers JC, Prentice AM, Stephenson J, Barker M, Saffery R, Yajnik CS, Eckert JJ et al Origins of lifetime health around the time of conception: causes and consequences. Lancet 2018;391:1842–1852. 10.1016/S0140-6736(18)30312-X PMC5975952 29673874

[deag125-B17] Flierman S , Tijsterman M, Rousian M, de Bakker BS. Discrepancies in embryonic staging: towards a gold standard. Life (Basel) 2023;13:1084. 10.3390/life13051084 PMC10222295 37240729

[deag125-B18] Graafland N , Rousian M, De Zwart M, Steegers-Theunissen R, Steegers E, Posthumus A. Parental conditions, modifiable lifestyle factors and first trimester growth and development: a systematic review. Hum Reprod Update 2025;31:166–182. 10.1093/humupd/dmaf001 PMC12046076 39953705

[deag125-B19] Graham JW , Olchowski AE, Gilreath TD. How many imputations are really needed? Some practical clarifications of multiple imputation theory. Prev Sci 2007;8:206–213. 10.1007/s11121-007-0070-9 17549635

[deag125-B20] Ioannou C , Sarris I, Hoch L, Salomon LJ, Papageorghiou AT; International Fetal and Newborn Growth Consortium for the 21st Century (INTERGROWTH-21st). Standardisation of crown-rump length measurement. BJOG 2013;120:38–41, v. 10.1111/1471-0528.12056 23678951

[deag125-B21] Jaddoe VWV , de Jonge LL, Hofman A, Franco OH, Steegers EAP, Gaillard R. First trimester fetal growth restriction and cardiovascular risk factors in school age children: population based cohort study. BMJ 2014;348:g14. 10.1136/bmj.g14 PMC3901421 24458585

[deag125-B22] Klonoff-Cohen H. Female and male lifestyle habits and IVF: what is known and unknown. Hum Reprod Update 2005;11:179–203. 10.1093/humupd/dmh059 15708968

[deag125-B23] Koning AH , Rousian M, Verwoerd-Dikkeboom CM, Goedknegt L, Steegers EA, van der Spek PJ. V-scope: design and implementation of an immersive and desktop virtual reality volume visualization system. Stud Health Technol Inform 2009;142:136–138. 19377132

[deag125-B24] Kooijman MN , Kruithof CJ, van Duijn CM, Duijts L, Franco OH, van IMH, de Jongste JC, Klaver CC, van der Lugt A, Mackenbach JP et al The Generation R Study: design and cohort update 2017. Eur J Epidemiol 2016;31:1243–1264. 10.1007/s10654-016-0224-9 PMC5233749 28070760

[deag125-B25] Li Y , Schoufour J, Wang DD, Dhana K, Pan A, Liu X, Song M, Liu G, Shin HJ, Sun Q et al Healthy lifestyle and life expectancy free of cancer, cardiovascular disease, and type 2 diabetes: prospective cohort study. BMJ 2020;368:l6669. 10.1136/bmj.l6669 PMC7190036 31915124

[deag125-B26] Loef M , Walach H. The combined effects of healthy lifestyle behaviors on all cause mortality: a systematic review and meta-analysis. Prev Med 2012;55:163–170. 10.1016/j.ypmed.2012.06.017 22735042

[deag125-B27] Mahendru AA , Daemen A, Everett TR, Wilkinson IB, McEniery CM, Abdallah Y, Timmerman D, Bourne T, Lees CC. Impact of ovulation and implantation timing on first-trimester crown–rump length and gestational age. Ultrasound Obstet Gynecol 2012;40:630–635. 10.1002/uog.12277 22858888

[deag125-B28] Mills JL , Holmes LB, Aarons JH, Simpson JL, Brown ZA, Jovanovic-Peterson LG, Conley MR, Graubard BI, Knopp RH, Metzger BE. Moderate caffeine use and the risk of spontaneous abortion and intrauterine growth retardation. JAMA 1993;269:593–597. 8421363

[deag125-B29] Mook-Kanamori DO , Steegers EA, Eilers PH, Raat H, Hofman A, Jaddoe VW. Risk factors and outcomes associated with first-trimester fetal growth restriction. JAMA 2010;303:527–534. 10.1001/jama.2010.78 20145229

[deag125-B30] O’Rahilly R , Müller F. Developmental Stages in Human Embryos: Including a Revision of Streeter’s “Horizons” and a Survey of the Carnegie Collection. Carnegie Institution of Washington, 1987.

[deag125-B31] O’Rahilly R , Müller F. Developmental stages in human embryos: revised and new measurements. Cells Tissues Organs 2010;192:73–84. 10.1159/000289817 20185898

[deag125-B32] Oostingh EC , Hall J, Koster MPH, Grace B, Jauniaux E, Steegers-Theunissen RPM. The impact of maternal lifestyle factors on periconception outcomes: a systematic review of observational studies. Reprod Biomed Online 2019;38:77–94. 10.1016/j.rbmo.2018.09.015 30424937

[deag125-B33] Oster H , Chaves I. Effects of healthy lifestyles on chronic diseases: diet, sleep and exercise. Nutrients 2023;15:4627. 10.3390/nu15214627 PMC10650398 37960280

[deag125-B34] Parisi F , Rousian M, Koning AHJ, Willemsen SP, Cetin I, Steegers-Theunissen RPM. Periconceptional maternal one-carbon biomarkers are associated with embryonic development according to the Carnegie stages. Hum Reprod 2017;32:523–530. 10.1093/humrep/dew349 28104698

[deag125-B35] Pietersma CS , Mulders A, Sabanovic A, Willemsen SP, Jansen MS, Steegers EAP, Steegers-Theunissen RPM, Rousian M. The impact of maternal smoking on embryonic morphological development: the Rotterdam Periconception Cohort. Hum Reprod 2022;37:696–707. 10.1093/humrep/deac018 PMC8971648 35193145

[deag125-B36] Pietersma CS , Mulders A, Willemsen SP, Graafland N, Altena AC, Koning AHJ, de Bakker BS, Steegers EAP, Steegers-Theunissen RPM, Rousian M. Embryonic morphological development is delayed in pregnancies ending in a spontaneous miscarriage. Hum Reprod 2023;38:820–829. 10.1093/humrep/dead032 PMC10152166 36966733

[deag125-B37] Prabhu N , Smith N, Campbell D, Craig LC, Seaton A, Helms PJ, Devereux G, Turner SW. First trimester maternal tobacco smoking habits and fetal growth. Thorax 2010;65:235–240. 10.1136/thx.2009.123232 20335293

[deag125-B38] Robinson HP. Sonar measurement of fetal crown-rump length as means of assessing maturity in first trimester of pregnancy. Br Med J 1973;4:28–31. 10.1136/bmj.4.5883.28 PMC1587065 4755210

[deag125-B39] Robinson HP , Fleming JE. A critical evaluation of sonar “crown-rump length” measurements. Br J Obstet Gynaecol 1975;82:702–710. 10.1111/j.1471-0528.1975.tb00710.x 1182090

[deag125-B40] Roos S , Kanai Y, Prasad PD, Powell TL, Jansson T. Regulation of placental amino acid transporter activity by mammalian target of rapamycin. Am J Physiol Cell Physiol 2009;296:C142–C150. 10.1152/ajpcell.00330.2008 18987252

[deag125-B41] Rosario FJ , Nathanielsz PW, Powell TL, Jansson T. Maternal folate deficiency causes inhibition of mTOR signaling, down-regulation of placental amino acid transporters and fetal growth restriction in mice. Sci Rep 2017a;7:3982. 10.1038/s41598-017-03888-2 PMC5479823 28638048

[deag125-B42] Rosario FJ , Powell TL, Jansson T. Mechanistic target of rapamycin (mTOR) regulates trophoblast folate uptake by modulating the cell surface expression of FR-α and the RFC. Sci Rep 2016;6:31705. 10.1038/srep31705 PMC4999868 27562465

[deag125-B43] Rosario FJ , Powell TL, Jansson T. mTOR folate sensing links folate availability to trophoblast cell function. J Physiol 2017b;595:4189–4206. 10.1113/JP272424 PMC5491891 28374905

[deag125-B44] Rousian M , Koster MPH, Mulders A, Koning AHJ, Steegers-Theunissen RPM, Steegers EAP. Virtual reality imaging techniques in the study of embryonic and early placental health. Placenta 2018;64:S29–S35. 10.1016/j.placenta.2018.01.001 29409677

[deag125-B45] Savitz DA , Terry JW Jr, Dole N, Thorp JM Jr, Siega-Riz AM, Herring AH. Comparison of pregnancy dating by last menstrual period, ultrasound scanning, and their combination. Am J Obstet Gynecol 2002;187:1660–1666. 10.1067/mob.2002.127601 12501080

[deag125-B46] Shipton D , Tappin DM, Vadiveloo T, Crossley JA, Aitken DA, Chalmers J. Reliability of self reported smoking status by pregnant women for estimating smoking prevalence: a retrospective, cross sectional study. BMJ 2009;339:b4347. 10.1136/bmj.b4347 PMC2771076 19875845

[deag125-B47] Silva E , Rosario FJ, Powell TL, Jansson T. Mechanistic target of rapamycin is a novel molecular mechanism linking folate availability and cell function. J Nutr 2017;147:1237–1242. 10.3945/jn.117.248823 PMC5483964 28592519

[deag125-B48] Stamatopoulos N , Lu C, Vo S, Vaquero ME, Leonardi M, Condous G. The effect of cigarette smoking on first-trimester crown–rump length. Australasian J Ultrasound Med 2022;26:21–25. 10.1002/ajum.12313 PMC10030090 36960140

[deag125-B49] Steegers-Theunissen RP , Twigt J, Pestinger V, Sinclair KD. The periconceptional period, reproduction and long-term health of offspring: the importance of one-carbon metabolism. Hum Reprod Update 2013;19:640–655. 10.1093/humupd/dmt041 23959022

[deag125-B50] Sterne JA , White IR, Carlin JB, Spratt M, Royston P, Kenward MG, Wood AM, Carpenter JR. Multiple imputation for missing data in epidemiological and clinical research: potential and pitfalls. BMJ 2009;338:b2393. 10.1136/bmj.b2393 PMC2714692 19564179

[deag125-B51] van der Windt M , Tobi EW, Chidi I, Schoenmakers S, van Rossem L, Steegers-Theunissen RPM, Rousian M. Periconceptional maternal and paternal alcohol consumption and embryonic and fetal development: the Rotterdam periconception cohort. Reprod Biomed Online 2024;49:104351. 10.1016/j.rbmo.2024.104351 39182451

[deag125-B52] Van Dijk MR , Borggreven NV, Willemsen SP, Koning AHJ, Steegers-Theunissen RPM, Koster MPH. Maternal lifestyle impairs embryonic growth: the Rotterdam Periconception Cohort. Reprod Sci 2018;25:916–922. 10.1177/1933719117728801 28884629

[deag125-B53] van Uitert EM , van der Elst-Otte N, Wilbers JJ, Exalto N, Willemsen SP, Eilers PH, Koning AH, Steegers EA, Steegers-Theunissen RP. Periconception maternal characteristics and embryonic growth trajectories: the Rotterdam Predict study. Hum Reprod 2013;28:3188–3196. 10.1093/humrep/det375 24105824

[deag125-B54] Verburg BO , Mulder PG, Hofman A, Jaddoe VW, Witteman JC, Steegers EA. Intra- and interobserver reproducibility study of early fetal growth parameters. Prenat Diagn 2008;28:323–331. 10.1002/pd.1972 18324617

[deag125-B55] Verwoerd-Dikkeboom CM , Koning AH, van der Spek PJ, Exalto N, Steegers EA. Embryonic staging using a 3D virtual reality system. Hum Reprod 2008a;23:1479–1484. 10.1093/humrep/den023 18296447

[deag125-B56] Verwoerd-Dikkeboom CM , Koning AHJ, Hop WC, Rousian M, Van Der Spek PJ, Exalto N, Steegers EAP. Reliability of three-dimensional sonographic measurements in early pregnancy using virtual reality. Ultrasound Obstet Gynecol 2008b;32:910–916. 10.1002/uog.5390 18792418

[deag125-B57] Voedingscentrum. Factsheet Cafeïne. Voedingscentrum, 2022.

[deag125-B58] von Elm E , Altman DG, Egger M, Pocock SJ, Gøtzsche PC, Vandenbroucke JP; STROBE Initiative. The Strengthening the Reporting of Observational Studies in Epidemiology (STROBE) statement: guidelines for reporting observational studies. Lancet 2007;370:1453–1457. 10.1016/S0140-6736(07)61602-X 18064739

[deag125-B59] Wassenaar S , Koch B. Direct biomarkers to determine alcohol consumption during pregnancy, which one to use? J Appl Bioanal 2015;1:76–79.

[deag125-B60] Weisskopf MG , Sparrow D, Hu H, Power MC. Biased exposure–health effect estimates from selection in cohort studies: are environmental studies at particular risk? Environ Health Perspect 2015;123:1113–1122. 10.1289/ehp.1408888 PMC4629739 25956004

[deag125-B61] Weuve J , Tchetgen Tchetgen EJ, Glymour MM, Beck TL, Aggarwal NT, Wilson RS, Evans DA, Mendes de Leon CF. Accounting for bias due to selective attrition: the example of smoking and cognitive decline. Epidemiology 2012;23:119–128. 10.1097/EDE.0b013e318230e861 PMC3237815 21989136

[deag125-B62] World Health Organization. Standards for Maternal and Neonatal Care. Geneva, Switzerland: World Health Organization, 2007.

[deag125-B63] World Health Organization. Guideline: Optimal Serum and Red Blood Cell Folate Concentrations in Women of Reproductive Age for Prevention of Neural Tube Defects. Geneva: World Health Organization, 2015. 25996016

[deag125-B64] Yung HW , Calabrese S, Hynx D, Hemmings BA, Cetin I, Charnock-Jones DS, Burton GJ. Evidence of placental translation inhibition and endoplasmic reticulum stress in the etiology of human intrauterine growth restriction. Am J Pathol 2008;173:451–462. 10.2353/ajpath.2008.071193 PMC2475782 18583310

