# Supplementary material for: Maternal lifestyle risk factors and human embryonic morphologic development using 3D ultrasound: a population-based prospective cohort study
Source: Hum Reprod. 2026 Aug 14;41(10):1703–14. doi: 10.1093/humrep/deag125 (PMC13635461; doi:10.1093/humrep/deag125)
Supplement: Supplementary file 1 [file deag125_supplementary_figure_s1.pdf]

Human Reproduction, 2026, 1–1
https://doi.org/10.1093/humrep/deag125
Supplementary Data

human reproduction
OXFORD

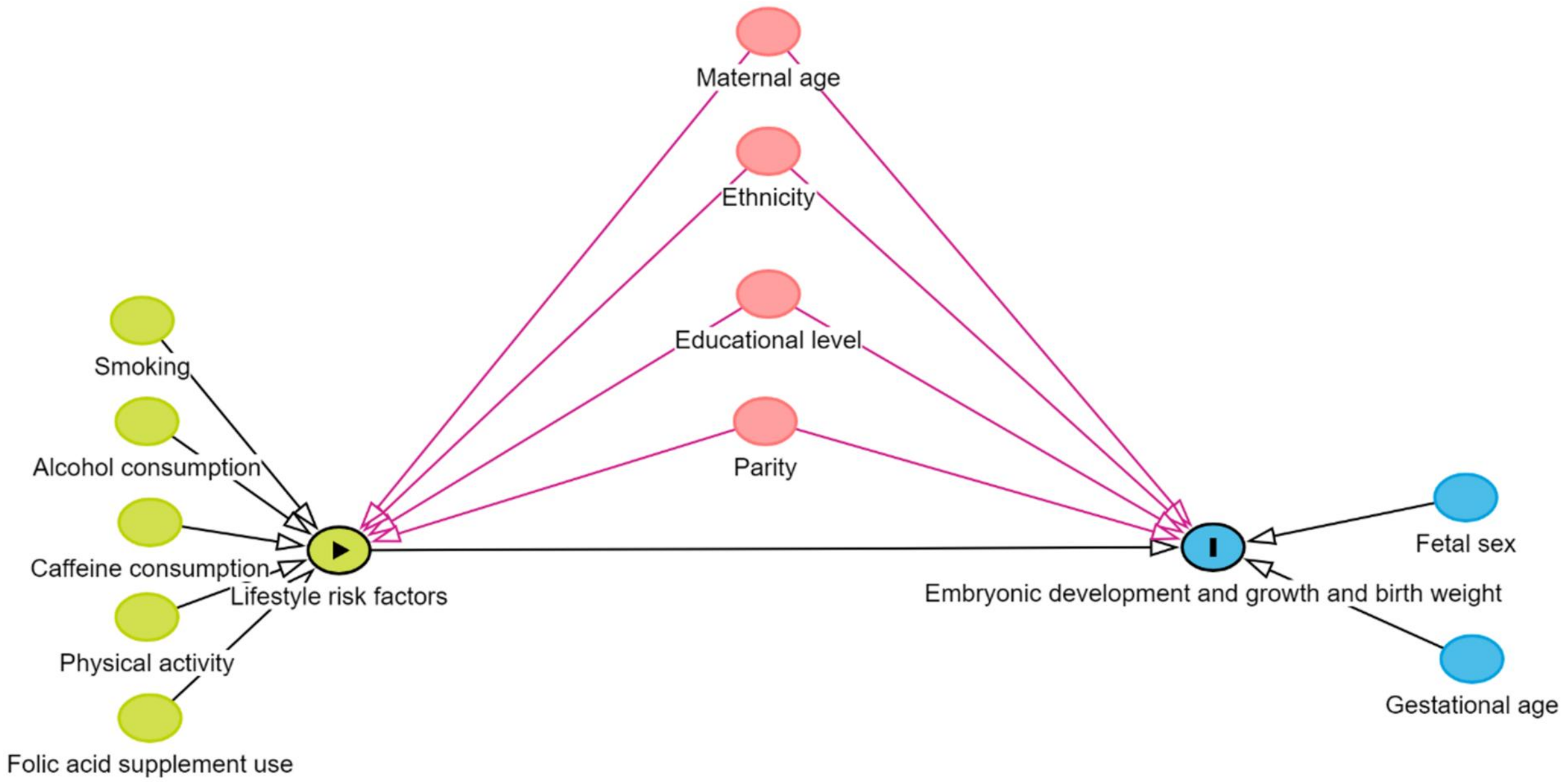

Supplementary Figure S1. Directed acyclic graph of lifestyle risk factors, embryonic development, and growth. Green nodes indicate exposure, blue nodes indicate outcomes, and red nodes indicate confounders. Black arrows represent the primary association of interest between lifestyle risk factors and embryonic development and growth/birth weight, whereas pink arrows represent potential confounding pathways.
