# Supplementary material for: Maternal lifestyle risk factors and human embryonic morphologic development using 3D ultrasound: a population-based prospective cohort study
Source: Hum Reprod. 2026 Aug 14;41(10):1703–14. doi: 10.1093/humrep/deag125 (PMC13635461; doi:10.1093/humrep/deag125)
Supplement: Supplementary file 2 [file deag125_supplementary_table_s1.pdf]

Human Reproduction, 2026, 1–1
https://doi.org/10.1093/humrep/deag125
Supplementary Data

human reproduction

OXFORD

Supplementary Table S1. Non-response analysis of participants included in and excluded from the study population.

Maternal characteristics	Included N = 993	Excluded[a] N = 612	*P*-value
Maternal age at conception (years)	31.4 (3.9)	30.3 (4.4)	<0.01
Missing	0	0	
Pre-pregnancy BMI (kg/m^2)	23.2 (21.2–26.0)	23.4 (21.0–26.9)	0.46
Missing	64	19 (3.9)	
Ethnicity (%)			<0.01
Dutch	665 (67.7)	294 (58.7)	
Non-Dutch, European	111 (11.5)	76 (15.2)	
Non-Dutch, non-European	202 (20.9)	131 (26.2)	
Missing	25 (2.5)	111 (18.1)	
Maternal educational level (%)			<0.01
Low	34 (3.5)	45 (9.1)	
Medium	201 (20.9)	142 (28.9)	
High	726 (75.6)	305 (62.0)	
Missing	32 (3.2)	120 (19.6)	
Monthly household income (%)			<0.01
<2000 euro	68 (7.7)	75 (17.5)	
2000–4000 euro	285 (32.2)	140 (32.6)	
>4000 euro	532 (60.1)	214 (49.8)	
Missing	108 (10.9)	183 (29.9)	
Parity (%)			<0.01
Nulliparous	627 (66.1)	271 (58.4)	
Parous	321 (33.9)	193 (41.6)	
Missing	45 (4.5)	148 (24.2)	
First trimester smoking (yes, %)	200 (22.2)	94 (21.8)	<0.01
Missing	90 (9.1)	180 (29.4)	
First trimester alcohol consumption (yes, %)	175 (18.3)	87 (18.7)	0.93
Missing	38 (3.8)	146 (23.9)	
Daily caffeine consumption, units[b] (%)			0.50
None	50 (5.4)	28 (6.9)	
<2 units	444 (48.2)	186 (45.7)	
2–3.9 units	348 (37.7)	163 (40.0)	
> 4 units	80 (8.7)	30 (7.4)	
Missing	71 (7.2)	205 (33.5)	
Physical activity (%)			0.10
Not active	17 (1.9)	13 (3.2)	
Moderately active (<150 min moderate or <75 min strenuous intensity)	211 (22.9)	118 (29.3)	
Very active (>150 min moderate or >75 min strenuous intensity)	693 (75.2)	272 (67.5)	
Missing	72 (7.3)	209 (34.2)	
Folic acid supplement use (inadequate, %)[c]	287 (30.5)	170 (40.1)	<0.01
Missing	52 (5.2)	188 (30.7)	
Serum folate (nmol/l)	37.7 (30.1–44.7)	34.9 (26.5–43.4)	<0.01
Sufficient[d] (≥25.5 nmol/l)	752 (85.3)	338 (70.7)	
Insufficient (<25.5 nmol/l)	129 (14.7)	140 (29.3)	
Missing	112 (11.3)	134 (21.9)	

Values are presented as mean (SD), median (IQR), or number of participants (%). Differences were tested by using the *t*-test, the Mann–Whitney *U*-test, or the χ^2 test.

[a] Participants excluded from the analyses had an unknown or unreliable LMP or an irregular menstrual cycle.

[b] One unit reflects 90 mg caffeine.

[c] Folic acid supplement use; adequate: preconceptional initiation of supplement use, inadequate: postconceptional initiation of supplement use or no use at all.

[d] Sufficient levels ≥25.5 nmol/l serum folate, based on recommendations for women of reproductive age.

IQR, interquartile range; LMP, last menstrual period.
