# Supplementary material for: Maternal lifestyle risk factors and human embryonic morphologic development using 3D ultrasound: a population-based prospective cohort study
Source: Hum Reprod. 2026 Aug 14;41(10):1703–14. doi: 10.1093/humrep/deag125 (PMC13635461; doi:10.1093/humrep/deag125)
Supplement: Supplementary file 3 [file deag125_supplementary_table_s2.pdf]

Human Reproduction, 2026, 1–1
https://doi.org/10.1093/humrep/deag125
Supplementary Data

human reproduction
OXFORD

Supplementary Table S2. Association between serum folate levels and embryonic development (Carnegie stages) and growth (crown-rump length), in a subset excluding participants with a serum folate above the upper LOD (>45.4 nmol/l) (N = 687).

	Carnegie stage (beta (95% CI)) *N = 626*		Crown-rump length (beta (95% CI)) *N = 640*	
	**Model 1**[a]	**Model 2**[b]	**Model 1**[a]	**Model 2**[b]
Serum folate (per IQR increase = 14.6 nmol/l)	0.27 (0.13–0.41)*	0.27 (0.12–0.43)*	0.73 (0.34–1.11)*	0.82 (0.38–1.26)*
Serum folate				
Sufficient (≥25.5 nmol/l)	*Reference*	*Reference*	*Reference*	*Reference*
Insufficient (<25.5 nmol/l)	−0.37 (−0.58 to −0.16)*	−0.33 (−0.57 to −0.10)*	−0.95 (−1.53 to −0.37)*	−0.98 (−1.62 to −0.34)*

[a] Model 1: GA.
[b] Model 2: GA, maternal age at conception, BMI, ethnicity, educational level, parity, smoking, and fetal sex.
* *P*-value <0.05.
LOD, limit of detection; IQR, interquartile range; GA, gestational age.

© The Author(s) 2026. Published by Oxford University Press on behalf of European Society of Human Reproduction and Embryology.
This is an Open Access article distributed under the terms of the Creative Commons Attribution License (https://creativecommons.org/licenses/by/4.0/), which permits unrestricted reuse, distribution, and reproduction in any medium, provided the original work is properly cited.
Human Reproduction, 2026, 00, 1–12
https://doi.org/10.1093/humrep/deag125
Original Article
